# Experimental Study on Effects of CO_2_ Curing Conditions on Mechanical Properties of Cement Paste Containing CO_2_ Reactive Hardening Calcium Silicate Cement

**DOI:** 10.3390/ma16227107

**Published:** 2023-11-09

**Authors:** Young-Jin Kim, Sang-Rak Sim, Dong-Woo Ryu

**Affiliations:** Department of Architectural Engineering, Daejin University, Pocheon-si 11159, Gyeonggi-do, Republic of Korea; rladud316@gmail.com (Y.-J.K.); simsr@daejin.ac.kr (S.-R.S.)

**Keywords:** CO_2_ curing, mineral carbonation, calcium silicate cement, CO_2_ sequestration, decalcification

## Abstract

Human survival is threatened by the rapid climate change due to global warming caused by the increase in CO_2_ emissions since the Second Industrial Revolution. This study developed a secondary cement product production technology by replacing cement, a conventional binder, with calcium silicate cement (CSC), i.e., CO_2_ reactive hardening cement, to reduce CO_2_ emissions and utilize CO_2_ from the cement industry, which emits CO_2_ in large quantities. Results showed that the carbonation depth, compressive strength increase rate, and CO_2_ sequestration rate increased as the CSC content increased, suggesting that CSC can be applied as a secondary cement product.

## 1. Introduction

Humanity is threatened by global warming due to the increase in CO_2_ emissions since the Second Industrial Revolution. Based on the “Global Warming 1.5 °C Special Report” from the Intergovernmental Panel on Climate Change, the average global temperature increased by approximately 1.09 °C after industrialization as of 2018, and the increase is expected to reach 1.5 °C between 2030 and 2052 [1]. This prompted the international community to establish the goal of 2050 carbon neutrality. To this end, carbon emissions should be effectively reduced, or high carbon emission reduction rates should be allocated to the energy conversion, waste, transport, and construction sectors, which generate high carbon emissions in the industrial sector [2].

In particular, the cement industry, which accounts for the majority of CO_2_ emissions from the mineral industry, is classified as a high-carbon-emitting industry [3]. Carbon emissions are increasing annually on a global scale. The 10 major countries contributing to carbon emissions are listed in Table 1 [4]. Carbon capture utilization (CCU) based on the carbonation reaction of cementitious materials has been presented as a key technology for CO_2_ reduction in the cement industry. In general, conventional concrete carbonation reactions have been recognized as a degradation factor that corrodes steel reinforcement because of the loss in alkalinity inside concrete caused by the carbonation of Ca(OH)_2_, which is a hydration product. However, studies have reported that proper carbonation reactions can improve strength and durability through the densification of the microstructure inside concrete [5]. Concrete is the second most consumed resource worldwide annually, and the implementation of efficient carbonation technology is expected to significantly reduce CO_2_ emissions from the cement industry [6,7].

**Figure 1:**
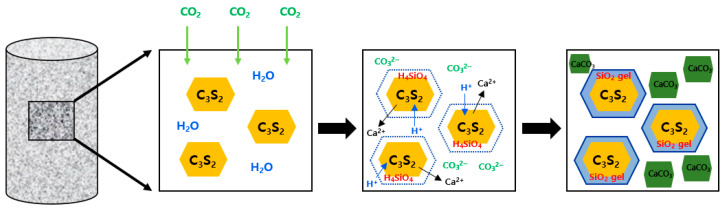
CSC curing mechanism.

To reduce CO_2_ emissions and utilize CO_2_ during the production and curing processes of cement, CO_2_ reactive hardening calcium silicate cement (CSC) has been extensively investigated. CSC primarily consists of rankinite (C_3_S_2_) and wollastonite (CS). It is non-hydraulic, as it does not react with water; however, it hardens through reactions with CO_2_, thereby developing strength [8]. The carbonation curing mechanism of CSC entails the penetration of CO_2_ into capillary pores during the initial stages of the reaction. This leads to the dissolution and ionization of water within the pores, resulting in the formation of H^+^, HCO^3−^, and CO_3_^2−^ ions. Specifically, the generation of significant quantities of H^+^ decreases the pH of the pore water and triggers the dissolution of Ca^2+^ from the C_3_S_2_ and CS phases. Then, H_4_SiO_4_ is polymerized to form a SiO_2_ gel with a high degree of polymerization. During the subsequent process of dissolution and polymerization, a slow depletion of H^+^ ions occurs, leading to the restoration of the pH of the pore solution. This creates an environment that is conducive to the formation of CaCO_3_. The formation of the SiO_2_ gel and CaCO_3_ around the C_3_S_2_ and CS phases while curing due to the above-mentioned reaction is the carbonation curing mechanism of CSC (see Figure 1) [9].

CSC production can reduce CO_2_ emissions by up to 70% compared with ordinary Portland cement (OPC) production owing to the reduced consumption of limestone and the reduced firing temperature (1250 °C). In addition, CO_2_ reduction due to the additional CO_2_ sequestration resulting from the carbonation curing process of secondary cement products can be expected [10,11].

Similarly, Bao Lu et al. [12] formulated a clinker that mostly comprises C_3_S_2_ and γ-C_2_S mineral phases to realize a cement clinker with low carbon content. They subjected it to carbonation curing and observed a direct correlation between the amount of CO_2_ fixation and the increase in compressive strength. Ashraf et al. [13] found that the highest amount of CaCO_3_ produced during the maximum carbonization of CSC is approximately 40% (by weight of paste).

In this study, a paste was prepared with different CSC contents and CO_2_ curing conditions, and basic mechanical properties were examined for the production of CSC-based secondary wet cement products for realizing CCU. CSC is non-hydraulic and develops strength through carbonation reactions. Because demolding is essential for the application of homogeneous carbonation curing to the entire surface of the paste containing CSC, blast-furnace slag cement (BSC) was added as a binder instead of OPC for minimum strength development. To realize CSC-based secondary cement products, strength should be developed for efficient demolding in the typical secondary cement product production process. Moreover, the optimal carbonation curing conditions and optimal CSC content that enables the development of strength equal to or higher than the required strength for conventional secondary cement products should be derived.

## 2. Experimental

### 2.1. Experimental Factors and Levels

The experimental factors and levels are presented in Table 2. The 0, 50, and 100 wt% BSC were replaced with CSC for cement paste. The corresponding specimens were labeled as P-CSC0, P-CSC50, and P-CSC100, respectively. Paste specimens with dimensions of 50 × 50 × 50 mm^3^ were prepared for the experiment.

### 2.2. Materials

**Figure 2:**
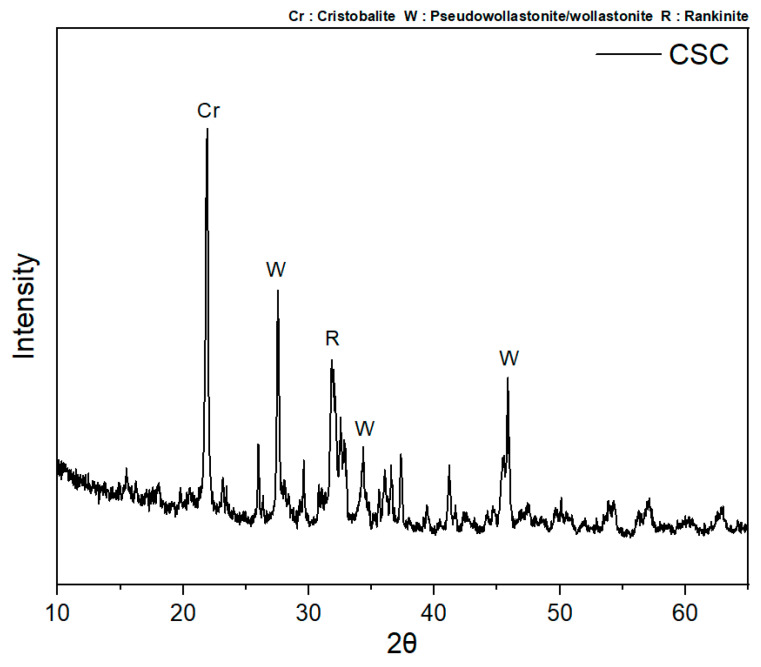
Main mineral facies of CSC.

In this study, BSC and CSC, which are commonly used for the production of secondary cement products and suitable for KS L 5210, from the company H (Seoul, Republic of Korea) were used. The chemical compositions of the materials were analyzed using X-ray fluorescence. The results are presented in Table 3. The primary mineral facies of CSC were analyzed using X-ray diffraction (XRD, Rigaku D, max 2200, Tokyo, Japan), as shown in Figure 2. A carbonation curing period of less than 24 h was applied considering the secondary product production process. The cement paste was analyzed, excluding the influence of the aggregate, to identify the hardening characteristics of cement containing CSC based on the carbonation reaction products.

### 2.3. Curing Method

Regarding the curing of the secondary wet cement products, after steam curing was performed as pre-curing before demolding, carbonation curing was applied uniformly to all the surfaces, excluding the bottom surface, of each specimen within 24 h after demolding.

**Figure 3:**
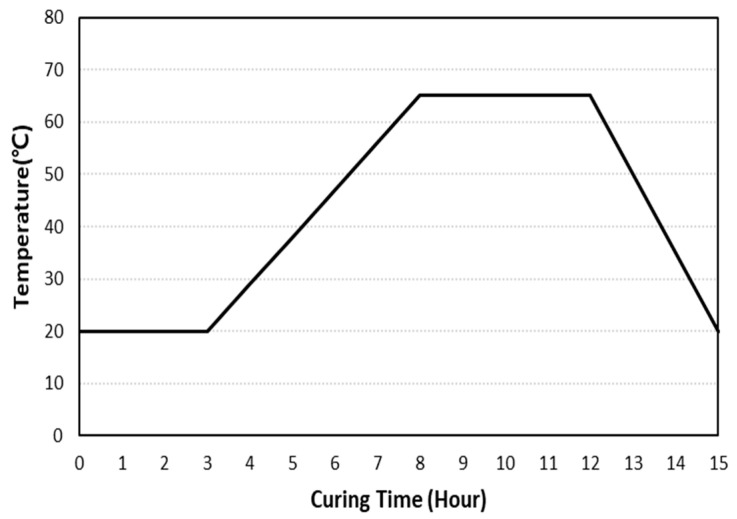
Steam curing method.

Steam curing was performed following KS L 4002 (empty concrete block). The accumulated temperature was set to 500 °C∙h by applying a pre-curing temperature of 20 °C, a maximum temperature of 65 °C, and a heating rate of 20 °C/h (Figure 3). For carbonation curing, CO_2_ concentrations of 20% and 99% and curing periods of 4, 8, 12, and 24 h were applied.

### 2.4. Test Methods

#### 2.4.1. Compressive Strength Evaluation

To evaluate the compressive strength based on the carbonation curing conditions, a test was conducted following KS L 5105 (compressive strength test method for hydraulic cement mortar). Specimens with dimensions of 50 × 50 × 50 mm^3^ were prepared. The compressive strength was measured for three samples upon completion of carbonation curing (4, 8, 12, and 24 h) at CO_2_ concentrations of 20% and 99%, and the average value was used.

#### 2.4.2. Carbonation Depth Evaluation

**Figure 4:**
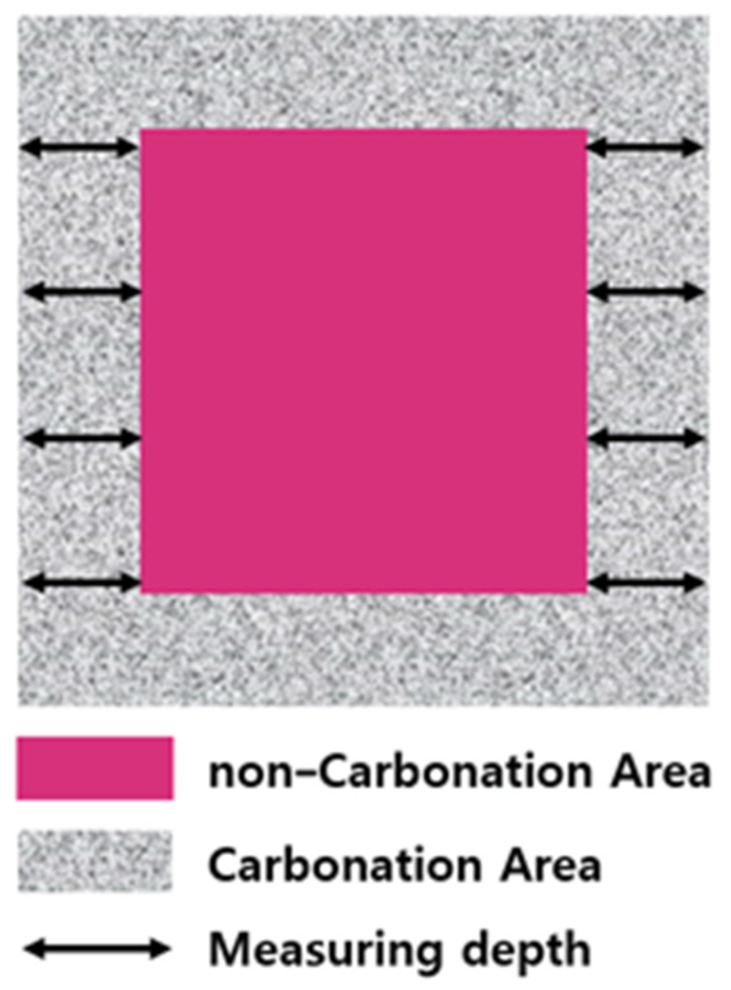
Carbonation depth measurement.

To evaluate the carbonation degree of paste containing CSC, 1% phenolphthalein solution was sprayed on the surface of each carbonation-cured specimen. The carbonation depth was measured at four left and right positions at equal intervals in areas that were not discolored, and the average value was calculated, as shown in Figure 4.

### 2.5. Quantitative Analysis

For quantitative analysis, 5 mm thick cubic samples were collected at depths of 5, 10, and 15 mm from the surface of each specimen using a microsampling cutter upon completion of carbonation curing. The collected samples were immersed in acetone solution for 2 d to stop hydration. They were crushed to a size of less than 75 µm, and the crushed powder was stored in a vacuum desiccator to maintain completely dry conditions.

#### 2.5.1. XRD Analysis

XRD analysis was conducted by collecting samples at depths of 5, 10, and 15 mm from the surface of each specimen. Measurements were performed using a tube current of 30 mA and a voltage of 40 kV. The XRD analysis was conducted in the scanning angle range of 5–65°.

#### 2.5.2. Thermogravimetric Analysis (TGA)

Thermogravimetry/differential thermal analysis (TG-DTA, STA 2500, Netzsch, Selb, Germany) was conducted to analyze the carbonation rate. Each processed powder was placed in a TGA sample holder, and the weight reduction rate was measured in the temperature range of 25–1000 °C at a heating rate of 10 °C/min in a high-purity nitrogen (99.999%) environment. For the main weight reduction of carbonated cement paste containing CSC, the weight reduction rate caused by the pyrolysis of the reaction products of BSC and CSC was measured.

#### 2.5.3. Fourier Transform Infrared Spectroscopy (FT-IR)

In an FT-IR (ALPHA2, Bruker, Ettlingen, Germany) analysis, scanning was performed in the wavenumber range of 400–4000 cm^−1^ with a resolution of 2 cm^−1^ in the attenuated total internal reflection mode for the samples collected near a depth of 5 mm from the surface.

Typical carbonation reactions of cement generate CaCO_3_ and silica gel by inducing the carbonation of Ca(OH)_2_ and C-S-H, which are hydration products. The carbonation reaction of Ca(OH)_2_ can promote the strength improvement effect by filling pores inside the hardened body, but the carbonation of C-S-H, which is responsible for strength, may reduce the strength through the contraction caused by decalcification [14,15,16,17]. To identify the carbonation degree of C-S-H generated by BSC, which is the binder of cement paste containing CSC, carbonation characteristics based on the carbonation period were analyzed using FT-IR spectroscopy, as this method can measure C-O bonds and Si-O chain structures.

## 3. Results

### 3.1. Carbonation Depth Analysis Results

**Figure 5:**
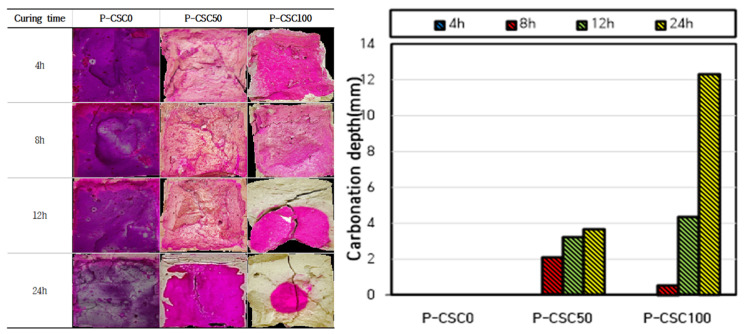
CO_2_ concentration 20% curing carbonation depth measurement result.

**Figure 6:**
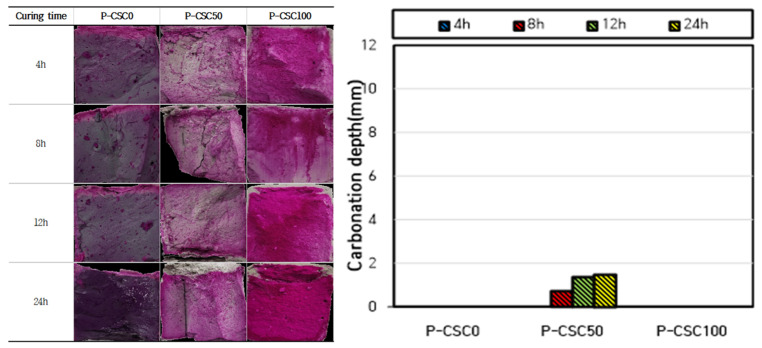
CO_2_ concentration 99% curing carbonation depth measurement result.

The carbonation depth measurement results are presented in Figure 5 and Figure 6. When carbonation curing was performed at a CO_2_ concentration of 20%, the carbonation depth tended to increase as the CSC content increased. When carbonation curing was performed for 24 h, a carbonation depth of 12.3 mm was observed for the P-CSC100 specimen. However, almost no carbonation occurred in the P-CSC0 (0 mm) and P-CSC50 (3.63 mm) mixes. For the P-CSC0 mix, no carbonation occurred because the penetration of CO_2_ was obstructed by the dense pore structure, as a high compressive strength of 55 MPa was developed by performing steam curing as pre-curing [18].

In addition, the carbonation depth rapidly increased in certain sections when carbonation curing was performed. The carbonation depth rapidly increased after 12 h of curing for the P-CSC50 mix and 8 h of curing for the P-CSC100 mix. This is because the diffusion of CO_2_ was reduced at the beginning of carbonation curing by the pores in the specimen saturated with moisture [19], as carbonation curing was performed immediately upon completion of steam curing under a 100% relative humidity condition as pre-curing. However, the carbonation depth was increased by the increased diffusion rate of CO_2_ along with the evaporation of moisture.

When the carbonation depth was measured for the specimens subjected to carbonation curing at a CO_2_ concentration of 99%, the P-CSC0 mix exhibited the same carbonation depth as that cured at a CO_2_ concentration of 20%. The highest carbonation depth was observed for the P-CSC50 mix, but it was as low as 0–2 mm. Moreover, the depth of carbonation could not be determined for the P-CSC100 mix. This is because the diffusion of CO_2_ decreased as micropores were filled with CaCO_3_, which was generated by the rapid carbonation of the specimen surface at the beginning of the reaction due to high-concentration CO_2_ diffusion [20].

### 3.2. Results of XRD Analysis and TG-DTA

**Figure 7:**
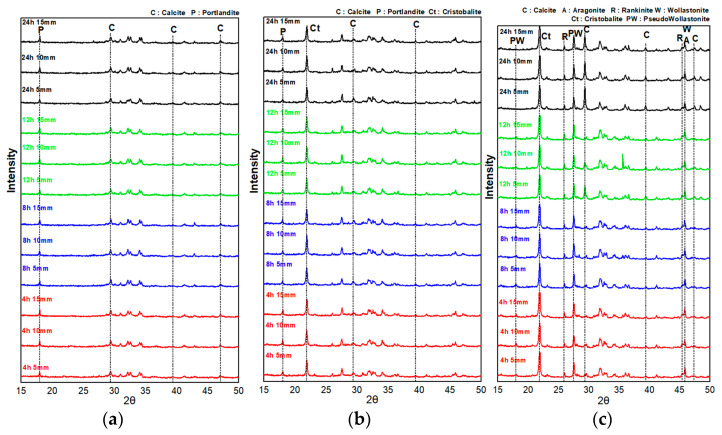
CO_2_ concentration 20% curing XRD pattern: (**a**) P-CSC0, (**b**) P-CSC50, and (**c**) P-CSC100.

Figure 7 shows the XRD patterns of the specimens collected at depths of 5, 10, and 15 mm subjected to carbonation curing at a CO_2_ concentration of 20%. Table 4 presents the TG-DTA results at positions where the carbonation depth was measured by spraying the phenolphthalein solution.

In the XRD analysis results for the CO_2_ concentration of 20%, the P-CSC0 mix exhibited Ca(OH)_2_ and calcite peaks. The Ca(OH)_2_ peak intensity decreased and the calcite peak intensity tended to slightly increase toward the surface. However, no significant peak change with the curing period was observed. Additionally, the TG-DTA measurement results indicated that the weight reduction rate tended to slightly increase as the carbonation curing period increased in the range of 600–800 °C, which is the pyrolysis range of CaCO_3_.

For the P-CSC50 and P-CSC100 mixes containing CSC, the peaks of pseudowollastonite, wollastonite, rankinite, and cristobalite, which are minerals that constitute CSC, and those of calcite and aragonite, which are carbonate minerals, were observed [13,21]. The XRD analysis results for the P-CSC50 mix indicated that the intensities of the Ca(OH)_2_ and calcite peaks were reduced and increased, respectively, after 8 h of carbonation curing. In the TG-DTA results for the samples of the 8, 12, and 24 h cured specimens collected near a depth of 5 mm from the surface, weight reduction rates of 4.23, 5.31, and 7.2 wt% were observed in the temperature range of 600–800 °C, respectively, confirming that the amount of CaCO_3_ generated increased as the carbonation curing period increased, in agreement with the XRD analysis results.

The XRD analysis results showed that, for the P-CSC100 mix, no significant formation of carbonate minerals occurred until 8 h of carbonation curing. However, the intensity of the calcite peak tended to rapidly increase after 12 h. For the specimens subjected to carbonation curing for 24 h, an increase in the calcite peak intensity for the specimen collected at a depth of 15 mm was confirmed. When TG-DTA was conducted using samples collected at a depth of 5 mm from the surface of the specimens subjected to carbonation curing for 4 and 8 h, weight reduction rates of 1.78 and 0.84 wt% were observed in the temperature range of 600–800 °C, respectively, indicating no carbonation. A high weight reduction rate of 11.39 wt% was observed for the 5 mm samples of the specimens subjected to carbonation curing for 12 h, and weight reduction rates of 12.04 to 9.82 wt% were observed for the 5–15 mm samples of the specimens subjected to carbonation curing for 24 h, confirming the occurrence of carbonation.

**Figure 8:**
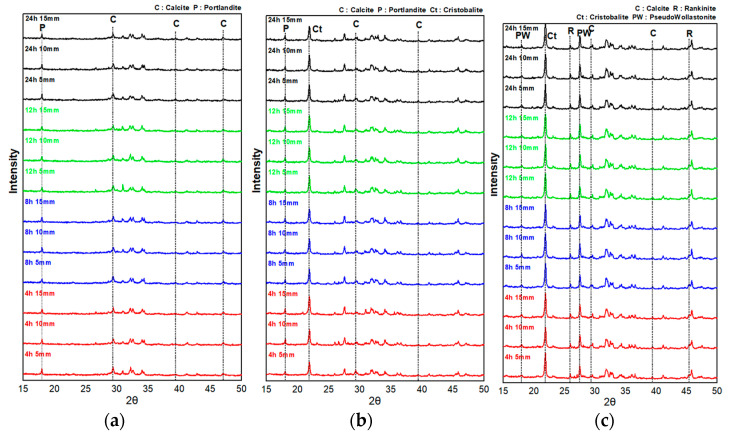
CO_2_ concentration of 99% curing XRD pattern: (**a**) P-CSC0, (**b**) P-CSC50, and (**c**) P-CSC100.

Figure 8 shows the XRD analysis results by depth for the P-CSC0, P-CSC50, and P-CSC100 paste specimens subjected to carbonation curing (4, 8, 12, and 24 h) at a CO_2_ concentration of 99%. Table 4 presents the weight reduction rate measurement results for the temperature ranges of 400–550 °C and 600–800 °C obtained via TG-DTA.

In the XRD analysis results, peaks of mineral facies similar to those of the specimens of the same mixes carbonation-cured at a CO_2_ concentration of 20% were observed. In contrast to the specimens cured at a CO_2_ concentration of 20%, for which the intensities of the peaks of carbonate minerals increased with the carbonation curing period, all the specimens cured at a CO_2_ concentration of 99% exhibited slight increases in the intensities of the peaks of carbonate minerals only at a depth of 5 mm from the surface when carbonation curing was performed for 8 h. Despite the increase in the carbonation curing period, no further changes in the intensities of the peaks of carbonate minerals were observed.

Additionally, the TG-DTA results showed that the weight reduction rate of CaCO_3_ increased slightly only at a depth of 5 mm from the surface after 8 h of carbonation curing in the 600–800 °C range, which is the pyrolysis temperature range of CaCO_3_. Despite the increase in the carbonation curing period, no carbonation was confirmed, as no change in the weight reduction rate was observed.

Thus, as discussed in Section 3.1, the quantitative XRD analysis and TG-DTA indirectly showed that the CO_2_ diffusion rate was reduced by the pores inside the hardened body saturated with moisture at the beginning of the carbonation curing (4 h) and that the diffusion rate of CO_2_ decreased as micropores were filled with CaCO_3_, which was generated by the rapid carbonation of the specimen surface due to high-concentration CO_2_ diffusion, after 8 h of carbonation curing.

### 3.3. FT-IR Analysis Results

**Figure 9:**
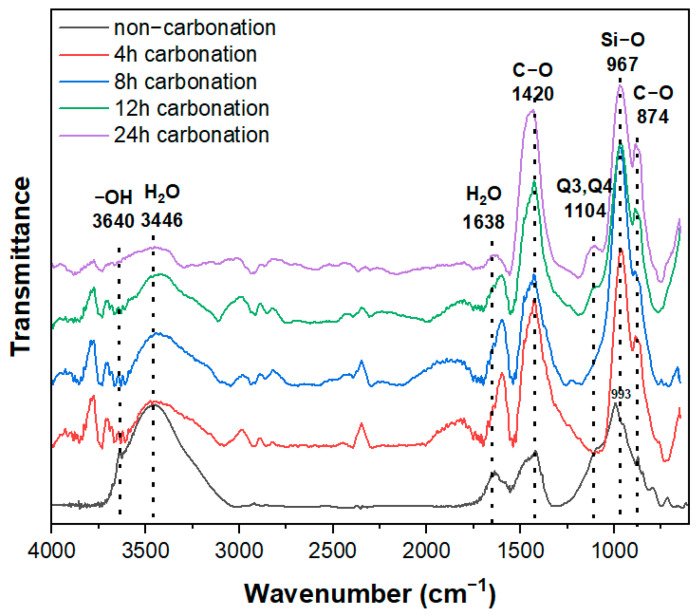
FT-IR analysis results of 20% concentration CO_2_-cured CSC0.

Figure 9 shows the FT-IR analysis results for P-CSC0 subjected to carbonation curing at a CO_2_ concentration of 20%. A peak at 3645 cm^−1^ corresponding to Ca(OH)_2_, a cement hydration product, was observed for the non-carbonated sample of the P-CSC0 mix [22]. However, the intensity of the Ca(OH)_2_ peak decreased after 12 h of carbonation curing and was not observed at 24 h of carbonation curing. The specimens subjected to carbonation curing exhibited rapid increases in the intensities of the peaks at 874 and 1420 cm^−1^ after 4 h of curing. These peaks suggested that CaCO_3_ was generated by C-O stretching [23]. The C-O stretching peaks indicated the generation of CaCO_3_ caused by the carbonation of Ca(OH)_2_. The carbonation was natural, as peaks at 874 and 1420 cm^−1^ were also observed for non-carbonated specimens, although they were weak compared with those of the carbonated specimens.

The peaks at 993 and 967 cm^−1^ for the non-carbonated and carbonated specimens corresponded to the Si-O stretching vibration of the Q^2^ site [22]. These peaks are observed for hydrated cement paste containing C-S-H. Their positions depend on the Ca/Si ratio, and their intensities are increased by the decalcification of C-S-H during the carbonation process [24]. When C-S-H is completely carbonated, the peak of Q^2^ moves to 1020 and 1140 cm^−1^, corresponding to the peaks of Q^3^ and Q^4^, respectively (silica gel). The P-CSC0 specimen exhibited a peak at 1104 cm^−1^ after 12 h of carbonation curing, confirming the decalcification of C-S-H.

### 3.4. Compressive Strength Analysis Results

**Figure 10:**
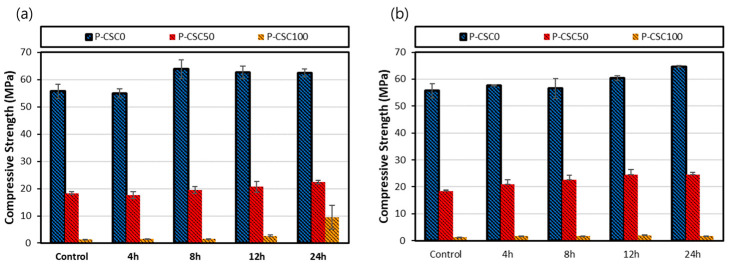
Compressive strengths results: (**a**) CO_2_ 20% and (**b**) CO_2_ 99%.

Figure 10 shows the compressive strength measurement results for the paste containing CSC. The compressive strengths measured immediately upon the completion of steam curing were 55.75, 18.34, and 1.37 MPa for CSC contents of 0%, 50%, and 100%, respectively, indicating that the strength decreased as the CSC content increased.

For the P-CSC0 mix, no significant change in compressive strength after 4 h of carbonation curing at a CO_2_ concentration of 20% was observed. The compressive strength was increased by approximately 9 MPa after 8 h and was reduced by approximately 1.2 MPa after 12 h compared with 8 h. Thereafter, no strength changes were observed. Notably, the compressive strength increased after 8 h of carbonation curing owing to the densification of the structure caused by the generation of CaCO_3_, as confirmed by the TG-DTA results. The strength was reduced after 12 h of carbonation curing because of the decalcification of C-S-H caused by an increase in the intensity of the 1104 cm^−1^ peak corresponding to silica gel (Q3 and Q4), as shown in Figure 6. Meanwhile, at a CO_2_ concentration of 99%, the compressive strength increased after 4 h of carbonation curing, and the generation of CaCO_3_ was confirmed by the TG-DTA results. The strength slightly decreased and then increased after 8 h of carbonation curing. This occurrence may be similar to the result for the 20% CO_2_ concentration.

The compressive strength of the P-CSC50 mix after carbonation curing was measured to be within the range of 17.62–22.5 MPa at a CO_2_ concentration of 20% and 20.89–24.56 MPa at a CO_2_ concentration of 99%. When the CO_2_ concentration was 20%, the compressive strength was 2 MPa lower on average than when it was 99%, despite the increase in carbonation depth. This difference is because mechanical properties deteriorated when sufficient CO_2_ diffusion into a deep part of concrete occurred compared with the case where carbonation occurred only in the surface layer because of the increase in the carbonation area of C-S-H (decalcification) [25]. At a CO_2_ concentration of 99%, however, the formation of a dense CaCO_3_ layer on the surface suppressed the diffusion of CO_2_ into the inside, suppressed the internal CSC carbonation reaction, and reduced the size of the C-S-H decalcification section, thereby increasing the compressive strength.

The P-CSC100 mix exhibited a compressive strength of approximately 1.3 MPa immediately after steam curing, confirming that CSC had no hydration reaction. When it was subjected to carbonation curing at a CO_2_ concentration of 20%, its compressive strength gradually increased after 12 h and rapidly increased to approximately 9.5 MPa after 24 h. However, it exhibited no compressive strength development until 24 h of carbonation curing at a CO_2_ concentration of 99%. When the weight reduction rate of CaCO_3_ measured via TG-DTA was compared with the compressive strength based on the carbonation curing period, the weight reduction rate was confirmed after 12 h when strength development began and increased after approximately 24 h at a CO_2_ concentration of 20%. At a CO_2_ concentration of 99%, the weight reduction rate of CaCO_3_ was higher than that at a CO_2_ concentration of 20% at the beginning of the reaction (after 4 h to 8 h) but did not increase despite the increase in the carbonation curing period. This accounts for the absence of strength improvement.

**Figure 11:**
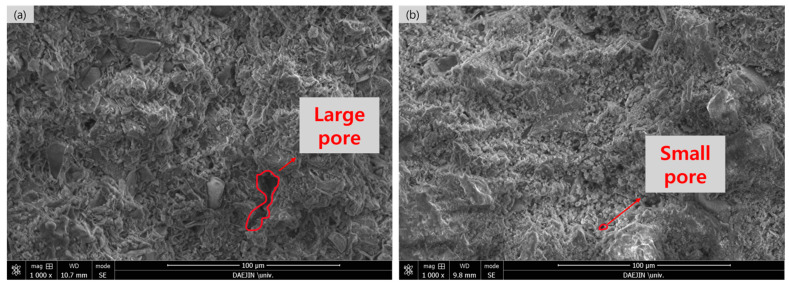
SEM images of 20% P-CSC50 specimens with CO_2_ concentration based on reaction time: (**a**) Carbonation curing time 4 h and (**b**) carbonation curing time 24 h.

In addition, SEM analysis was conducted on the P-CSC50 mix with a 20% CO_2_ concentration to determine the cause of the increase in compressive strength based on the carbonation curing period. Figure 11 shows a distinct disparity in the microstructure between the 4 and 24 h carbonation curing specimens. The 4 h curing specimen exhibited the formation of large pores, and the 24 h curing specimen exhibited a dense pore structure. However, the large pores observed in the 4 h specimen were not apparent.

## 4. Discussion

### 4.1. Effect of Moisture on Carbonation Curing

Moisture is essential for the carbonation reaction of typical concrete as CO_2_ and Ca^2+^ ions are dissolved in water for the reaction. However, the presence of a considerable amount of water in the concrete pores reduces the diffusion rate of CO_2_ to the order of 1/10,000 of that in gas, slowing the carbonation reaction [26,27].

Moradllo et al. [19] investigated the effect of water saturation on the carbonation of CSC mortars and found that carbonation did not occur even when carbonation curing was performed if the internal water saturation of the mortar was 100%. They reported that the carbonation rate increased approximately four times as the water saturation decreased from 80% to 10% [19].

When the carbonation depth was examined based on the above-described carbonation curing conditions, the carbonation depth rapidly increased under certain carbonation curing period conditions. This indicated that the diffusion of CO_2_ was hindered at the beginning of the carbonation curing by pores in the specimen saturated with moisture, as the carbonation curing was performed immediately upon the completion of steam curing under the 100% relative humidity condition as pre-curing. However, the carbonation depth was increased by the increased diffusion rate of CO_2_ along with the evaporation of moisture. Thus, a drying process is necessary to increase the carbonation rate at the beginning of the carbonation reaction.

### 4.2. Changes in Mechanical Properties during Carbonation Curing

The typical carbonation reactions of BSC generate CaCO_3_ and silica gel by inducing the carbonation of Ca(OH)_2_ and C-S-H, which are hydration products. The carbonation reaction of Ca(OH)_2_ can promote the strength improvement effect by filling the pores inside the hardened body, but the carbonation of C-S-H, which is responsible for strength, may reduce the strength through the contraction caused by decalcification. Therefore, in this study, before the evaluation of mechanical properties after carbonation curing, we assumed that CSC would be hardened by the carbonation reactions of CS and C_3_S_2_ and that the mechanical properties of BSC would be changed by the carbonation reactions of the hydration products generated by steam curing. In the compressive strength analysis results based on this, the strength improvement effect was offset by the decalcification of C-S-H, which is a BSC-based hydration product, in sections where the compressive strength decreased despite the increase in the amount of CaCO_3_ due to carbonation curing. Therefore, the proper carbonation reaction conditions should be derived considering both the changes in mechanical properties caused by the carbonation reaction of the hydration products of BSC and the mechanical property improvement due to the carbonation reaction products of CSC.

## 5. Conclusions

In this study, the basic mechanical properties and CO_2_ sequestration rate of paste were evaluated based on the CSC content and carbonation curing conditions to develop secondary cement products containing CSC for CO_2_ reduction. The optimal CSC content and carbonation curing condition were determined.

The carbonation depth results for cement paste with different CSC contents indicated that the carbonation depth tended to increase as the CSC content increased. Moreover, the carbonation depth rapidly increased in certain sections. This appears to be because the diffusion rate of CO_2_ was reduced at the beginning of carbonation curing by the pores in the specimen saturated with moisture, as the carbonation curing was performed immediately upon the completion of steam curing, or the carbonation depth was increased by the increased diffusion rate of CO_2_ along with the evaporation of moisture. The carbonation depth was higher for the 20% CO_2_ concentration curing condition than for the 99% CO_2_ concentration curing condition. This appears to be because the diffusion rate of CO_2_ decreased as micropores were filled with CaCO_3_, which was rapidly generated on the specimen surface by the high-concentration CO_2_ at the beginning of the carbonation reaction.

The compressive strength results for paste containing CSC indicated that the compressive strength tended to increase after carbonation curing. However, for the P-CSC0 mix, strength degradation sections were observed. FT-IR analysis results indicated that these sections were caused by the decalcification of C-S-H, which is a BSC-based hydration product. Accordingly, for the P-CSC50 mix, CSC was expected to increase the strength through carbonation, but the strength improvement effect was offset by the decalcification of C-S-H. For the P-CSC100 mix, no strength improvement at a CO_2_ concentration of 99% was observed, in contrast to the case of a CO_2_ concentration of 20%.

This study conducted a basic investigation to develop secondary products of cement utilizing CSC to advance the CCU technology in the cement industry. Furthermore, the application of CSC is a technological approach that can effectively decrease CO_2_ emissions throughout the cement production process while utilizing CO_2_. It is expected to serve as a direct solution for CO_2_ reduction and contribute to the advancement of CCU technology in the cement industry.

## Figures and Tables

**Table 1:** Carbon emissions per capita in major countries [4].

Country	CO_2_ Emissions per Capita (tCO_2_)	CO_2_ Emissions (MtCO_2_)
China	8.0	11,472.37
United States of America	15	5007
India	1.9	2710
Russian Federation	12	1756
Japan	8.6	1067
Iran	8.5	749
Germany	8.1	675
Saudi Arabia	19	672
Indonesia	2.3	619
South Korea	12	616

**Table 2:** Experimental factors and levels.

Factors	Title 2		Levels	Notes
Specimens	W/B		0.36	Paste
	CSC (%)		0, 50, 10	
Curing	Pre-curing		Steam curing	KS L 4004
	Carbonation Curing	CO_2_ 20%	4, 8, 12, 24 h	
		CO_2_ 99%	4, 8, 12, 24 h	
Mechanical properties			Compressive strength	KS F 5105
Quantitative analysis			Carbonation depth, XRD, TG-DTA, FT-IR, SEM	

**Table 3:** Chemical composition of binder (obtained by X-ray fluorescence spectroscopy (Rigaku D, ZSX PrimuslV, Tokyo, Japan)).

Chemical Composition (wt%)					
	CaO	SiO_2_	Al_2_O_3_	Fe_2_O_3_	MgO
BSC	52.64	19.86	7.41	2.21	4.2
CSC	48.28	44	0.45	0.67	0.46

**Table 4:** TG-DTA results.

Title 1		CO_2_ 20%		CO_2_ 99%	
		400–500 °C	600–800 °C	400–500 °C	600–800 °C
CSC0	4 h—5 mm	1.48	1.55	1.36	2.76
	8 h—5 mm	1.63	2.36	1.53	3.05
	12 h—5 mm	1.23	2.76	1.2	2.3
	24 h—5 mm	1.54	2.04	1.23	2.36
CSC50	4 h—5 mm	1.04	3.64	1.45	3.32
	8 h—5 mm	1.24	4.23	1.68	2.72
	12 h—5 mm	0.63	5.31	1.06	3.04
	24 h—5 mm	0.95	7.2	1.23	3.02
CSC100	4 h—5 mm	1.17	1.78	0.88	2.57
	8 h—5 mm	0	0.84	0.53	4.13
	12 h—5 mm	0	11.39	1.55	1.26
	24 h—5 mm	0.16	12.04	1.6	1.36
	24 h—10 mm	0.17	11.05	1.2	1.02
	24 h—15 mm	0.13	9.04	1.16	0.97

## Data Availability

The data presented in this study are available upon request from the corresponding author.
